# Bridging the knowledge gap: a mixed-methods study on general practitioners’ information needs for mHealth apps in hypertension treatment in Germany

**DOI:** 10.1186/s12913-025-13192-9

**Published:** 2025-09-10

**Authors:** S. May, F. Seifert, D. Bruch, K. Voß, M. Heinze, S. Spethmann, F. Muehlensiepen

**Affiliations:** 1 Center for Health Services Research, Brandenburg Medical School Theodor Fontane, Seebad 82/83, Rüdersdorf, 15562 Germany; 2 Department of Cardiovascular Surgery, Heart Center Brandenburg, Faculty of Health Sciences, Brandenburg Medical School Theodor Fontane, Bernau Bei Berlin, 16321 Germany; 3 Deutsches Herzzentrum der Charité – Department of Cardiology, Angiology and Intensive Care Medicine, Charitéplatz 1, Berlin, 10117 Germany; 4 Charité – Universitätsmedizin Berlin, corporate member of Freie Universität Berlin and Humboldt-Universität Zu Berlin, Berlin, 10117 Germany; 5 Association of Statutory Health Insurance Physicians Brandenburg (“KVBB”), Potsdam, 14467 Germany; 6 Department of Psychiatry and Psychotherapy of the Brandenburg Medical School, Immanuel Klinik Rüdersdorf, Rüdersdorf, 15562 Germany; 7 AGEIS, Université Grenoble-Alpes, Grenoble, 38000 France

**Keywords:** Mixed-methods, MHealth apps, General practitioner, Hypertension management, Information needs

## Abstract

**Background:**

Hypertension remains a critical public health issue in Germany, affecting millions of individuals. Mobile health applications (mHealth apps) offer promising solutions for improving patient outcomes and adherence in hypertension management. Despite their advantages in healthcare, the adoption of mHealth apps by general practitioners (GPs) in Germany remains limited to date.

**Objective:**

This study aimed to explore the needs, attitudes, and recommendation behaviours of GPs regarding mHealth apps in hypertension management.

**Methods:**

A mixed-methods approach was utilised, starting with a quantitative questionnaire of 205 GPs in Berlin and Brandenburg to collect representative data on attitudes, information needs, and app recommendation behaviour. Two focus groups (*N* = 15) provided qualitative insights into the reasons behind observed behaviours and potential solutions for addressing information gaps.

**Results:**

Of the 205 participating GPs, 41.5% currently recommend mHealth apps, while 77.6% expressed a willingness to recommend them in the future. Positive attitudes toward mHealth apps were widespread, with 37.6% viewing their integration into hypertension management positively and 43.4% somewhat positively. Furthermore, 54.1% rated the usefulness of mHealth apps as high or very high, and 35.1% provided similar ratings for user-friendliness. However, 59.1% reported feeling poorly or not informed at all about mHealth apps for hypertension management. Positive attitudes significantly increased the likelihood of recommending mHealth apps (G = 0.955, *p* < 0.001), as did higher ratings for usefulness (G = 0.887, *p* < 0.001) and user-friendliness (G = 0.625, *p* < 0.001). Notably, a high need for information about app functionalities (G = 0.617, *p* < 0.001) and available app offerings (G = 0.710, *p* < 0.001) was strongly associated with willingness to recommend apps. The focus groups highlighted gaps in systematic information dissemination and practical integration of mHealth apps, with participants calling for targeted educational resources and improved technical support.

**Conclusion:**

While GPs in Berlin and Brandenburg are generally willing to integrate mHealth apps into hypertension management, information gaps remain. Providing tailored educational resources and fostering collaboration among stakeholders could improve the uptake and effective use of mHealth apps in practice. Further investigation is needed to understand the relationship between knowledge levels and recommendation behaviours.

**Trial Registration:**

Identifier DRKS00029761, Registration Date: 27.07.2022.

**Supplementary Information:**

The online version contains supplementary material available at 10.1186/s12913-025-13192-9.

## Introduction

Arterial hypertension is one of the most common chronic diseases and a leading risk factor for cardiovascular diseases [1, 2], affecting millions of people worldwide [1]. In Germany, more than one in four people with statutory health insurance (26.3%, 19 million) was diagnosed with high blood pressure in 2018 [3] and were treated primarily by General Practitioners (GP). Digital medical technologies are developing rapidly, particularly in the area of mobile health applications (mHealth apps). mHealth apps are well adopted by patients, can effectively lower blood pressure and significantly improve adherence to antihypertensive treatment [4, 5]. Consequently, they can play an important role in the prevention and treatment of hypertension [6], thereby demonstrating how digital innovations are revolutionising the care of people with chronic conditions and opening up new perspectives for more effective healthcare.

In Germany, there is a unique situation in which dedicated mHealth apps (in German: digitale Gesundheitsanwendungen (DiGA)) [7] can be prescribed by physicians and the costs are fully reimbursed by health insurance companies. To gain DiGA status, mHealth app providers must prove the safety, functionality, quality, data security, and basic effectiveness of their application through a clinical study [8]. Currently, there is only one ‘DiGA’ for the treatment of arterial hypertension, which was approved after the design of this study [9]. Despite these promising structural developments and the available evidence, the use and recommendation of mHealth apps by GPs in Germany is still relatively rare [10].

Several studies have investigated the acceptance of mHealth apps by GPs and their informational needs, shedding light on barriers to their broader adoption in clinical practice. For instance, Byambasuren et al. [11] highlighted that GPs are generally open to incorporating mHealth apps into patient care but feel underinformed about their specific capabilities and the supporting evidence base, emphasizing the need for more targeted educational initiatives [11]. Similarly, a study by Della Vecchia et al. [12] demonstrated that while many French GPs are willing to prescribe mHealth apps, they require clear guidelines and robust evidence of effectiveness to build confidence in their use [12]. In Germany, Wangler and Jansky [10] reported similar findings, with GPs expressing interest in mHealth apps but citing concerns about their practicality, data security, and the time required to evaluate and implement them in routine care [10].

Currently, there are no systematic studies on the use and acceptance of mHealth apps in primary care in rural healthcare settings, which is why this study focuses on the federal state of Brandenburg. Brandenburg, home to 2.5 million inhabitants, is characterized by a relatively low population density and an aging population, presenting specific challenges for healthcare delivery [13]. Limited availability of medical professionals, long travel distances to healthcare facilities, and associated structural care gaps highlight the potential relevance of digital healthcare solutions in this region [13–15]. Additionally, Brandenburg ranks among the German states with the highest prevalence of arterial hypertension, further underscoring the importance of innovative care solutions in this area [3]. In a rural region like Brandenburg, mHealth apps could play a significant role in improving patient outreach, mitigating care shortages, and enhancing adherence in the management of chronic diseases such as hypertension [10, 11].

The limited research that has been conducted on GP attitudes towards mHealth apps suggests that health professionals’ adoption of such technologies depends on several factors, including the perceived usefulness, ease of use and trustworthiness of the applications [16].

The aim of this study is to determine the current attitudes towards the use of mHealth apps in hypertension management and the recommendation behaviour of GP´s, as well as to analyse the existing need for information and to determine how this can be fulfilled in order to establish acceptance in daily care. The focus is on identifying the reasons for these deficits, analysing their impact on clinical practice, and outlining potential approaches to improve the availability of information.

## Methods

This study is part of the DiPaH-project (Digital preventive measures for arterial hypertension) [17], which examines structural and individual factors in patients with arterial hypertension and healthcare providers that influence the use of digital preventive measures in Germany. A mixed-methods approach (explanatory sequential design) was chosen to ensure a comprehensive understanding of the topic [18]. Firstly, a quantitative questionnaire was conducted to generate representative data and gain a broad overview of the most important topics. Building on the results of the questionnaire, an in-depth qualitative analysis was conducted using focus groups in order to contextualize the results and gain differentiated insights into the subjective perspectives of the participants. Data integration was conducted according to the approach described by Fetters et al. [19], where the results of the quantitative questionnaire informed the design of the qualitative focus groups, enabling a sequential and complementary integration of findings.

This study was approved by the Ethics Committee of the Brandenburg Medical School Theodor Fontane (E-02–20220620). This manuscript and its reporting are aligned with checklists for the Consolidated Criteria for Reporting Qualitative Research (COREQ) [20], the reporting of observational studies (STROBE) [21] and reporting of a mixed-methods study (GRAMMS) [22] (please see Supplementary Material 1, 2 and 3).

### Questionnaire

The cross-sectional, self-reported, online questionnaire was conducted over a 6-month period between November 2023 to March 2024 in cooperation with the Association of Statutory Health Insurance Physicians Brandenburg (in German: Kassenärztliche Vereinigung Brandenburg (KVBB)) with participating general practitioners (N = 205). Participants were selected based on the following inclusion criteria: (1) general practitioners, (2) practicing in health care, (3) based in Germany, and (4) active (i.e., not retired and not in training). The questionnaire investigated attitudes, information needs, potentials, and barriers to the implementation of mHealth apps in hypertension treatment. It was developed based on the results of the qualitative preliminary study with cardiologists, general practitioners and nurses [23]. The questionnaire was divided into the following sections:

- Using digital health technologies and digital preventive measures in current and future practice
- Information needs in the field of mHealth apps
- Attitudes towards mHealth apps
- Sociodemographic data (gender, age, duration of professional activity, number of inhabitants of the clinical location, setting, employment relationship and federal state)
- Potentials and barriers of mHealth apps in hypertension treatment (another publication on these results is planned and is not part of this paper)
- Vignettes on mHealth app recommendations (another publication on these results is planned and is not part of this paper)
- Digital health literacy (eHEALS) (another publication on these results is planned and is not part of this paper)

The questionnaire was pretested with ten colleagues and five physicians (cardiologists and general practitioners) to ensure questionnaires comprehensibility and clarity. The questionnaire was revised in several rounds of feedback. In total, the questionnaire was revised nine times until there were no more inaccuracies among the test persons. During this process, several refinements were made to improve the instrument’s clarity, relevance, and usability. These included linguistic adjustments for precision and gender-inclusive language, adaptation and relabeling of response scales to enhance interpretability, and restructuring of item blocks to ensure a more logical flow and reduce respondent burden. In addition, redundant or ambiguous items were removed, and new content was added to address innovation attitudes, information needs, and the use of apps in hypertension care (please see the final questionnaire in supplementary material file 4). The final questionnaire was offered in a paper and pencil version or online using UniPark, a web-based survey tool [24]. The KVBB contacted a total of 708 randomly selected GPs in Brandenburg. The GPs were able to complete and return the enclosed questionnaire directly or access the online questionnaire via a link and complete the questionnaire digitally. Participants received a compensation of EUR 50 for completing the questionnaire. Written informed consent was obtained after participants had been informed about the study.

The sample size was determined to be *n* = 200 based on an a priori power analysis (G*Power) for the χ^2^ test with the following assumptions: medium effect size (w = 0.2), α = 0.05, power (1-β) = 0.80, and df = 1. The required sample size is *n* = 197. Statistical analyses were performed using SPSS Statistics for Windows, version 23 (IBM Corp). In addition to the descriptive results, we conducted correlation analyses (according to Goodman and Kruskal's gamma). Goodman and Kruskal's gamma is a nonparametric statistic used to assess the strength and direction of association between two variables on an ordinal scale with many tied ranks [25]. For the analysis using Goodman and Kruskal's gamma, the response option'I cannot assess'was coded as missing regarding the user-friendliness of mHealth apps.

### Focus groups

The aim of the focus groups was to triangulate and enrich the questionnaire findings by providing complementary qualitative insights and deeper contextual understanding. Two focus groups (*N* = 15) were conducted in June 2024. The recruitment of participants in the focus groups followed an ad hoc voluntary approach. Persons, who participated in the questionnaire, have had the opportunity to leave their contact information. The participants were contacted by e-Mail and invited to participate in the focus groups. Furthermore, institutions collaborating with the DiPaH-project [17] were invited to participate in a focus group. The inclusion criterion for the focus groups was that participants were healthcare professionals or stakeholders in Germany who work in the treatment of people with hypertension or are involved in the prevention of hypertension. The focus groups were conducted online and began with the presentation of the questionnaire results followed by a discussion with participants (please see the interview guide in supplementary material file 5).

The focus groups were audio-recorded and transcribed verbatim. Qualitative analyses of the focus groups were performed iteratively by two health researchers (S.M., F.M.), based on Kuckartz’s structured qualitative content analysis [26] using MAXQDA software (Verbi GmbH version 20.0.8). An inductive coding approach was applied, with categories and subcategories developed directly from the data without the use of a predefined coding framework. First, quotes directed by the aim were extracted and condensed into codes. Main categories and subcategories were formed from the codes. Consensus discussions were held continuously in the research group until a common understanding of all the emerging categories was achieved. Data collection and analysis were circular and continued until no substantially new findings emerged and theoretical saturation was reached. Saturation was assessed iteratively during data collection and analysis. After the second focus group, no new relevant themes emerged and previously identified categories were consistently confirmed across both focus groups. In addition, the heterogeneity of the participant sample (including general practitioners, cardiologists, nurses, health insurance representatives, and app developers) ensured that a broad range of perspectives was captured in line with the study’s aims. For the presentation of the results, representative quotes from the transcripts were selected, translated into English, and included in the manuscript.

## Results

### Questionnaire

#### Participants characteristics

Out of the 708 GPs contacted, a total of 205 completed the questionnaire (response rate: 28%). Of the participants, 64.4% (132/205) participated via mail, whereas 35.6% (73/205) completed the questionnaire online. Mean age was 50.6 (SD 10.4) years; 57.1% (117/205) were female, 40.5% (83/205) were male, 0.5% (1/205) non-binary and 2.0% (4/205) did not give any information on gender. The study sample’s demographics are shown in Table 1.

**Table 1:** Detailed participants characteristics of the surveyed GP`s; *if practicing at multiple locations, GPs were asked to indicate the population of their primary practice location

	**Characteristics**	**Participants (*n* = 205)**	
		**n**	**%**
1	Years of professional activity as a general practitioner		
	< 10	78	38.0
	10–20	62	30.2
	21–30	32	15.6
	31–40	22	10.7
	> 40	7	3.4
	Missing	4	2.0
2	Gender		
	Female	117	57.1
	Male	83	40.5
	Non-binary	1	0.5
	Missing	4	2.0
3	Age (in years)		
	< 40	33	16.1
	40–50	68	33.2
	51–60	66	32.2
	61–70	29	14.1
	> 70	7	3.4
	Missing	2	1.0
4	Number of inhabitants of the location where the GP practice is located*		
	< 5.000	40	19.5
	5.000—20.000	86	42.0
	20.001—100.000	49	23.9
	100.001–1 Mio	16	7.8
	> 1 Mio	9	4.4
	Missing	5	2.4
5	Setting		
	Single handed practice	108	52.7
	Practice partnership	65	31.7
	Medical care centre	28	13.7
	Hospital	1	0.5
	Missing	3	1.5
6	Employment relationship		
	Self-employed	51	24.9
	Employed	149	72.7
	Self-employed and employed	3	1.5
	Missing	2	1.0

#### Recommendation of mHealth apps

In the questionnaire, 41.5% (85/205) of GPs reported currently recommending disease-specific mHealth apps (excluding DiGAs), such as those for hypertension, in their daily practice. In contrast, 53.7% (110/205) stated that they do not recommend such apps, and 3.4% (7/205) reported that they had previously recommended disease-specific apps but have since stopped doing so. The willingness to recommend disease-specific apps in the future is more promising: 77.6% (159/205) of respondents expressed that they could envision recommending disease-specific mHealth apps in the future, while only 16.1% (33/205) rejected the idea.

#### Attitudes, perceived usefulness, usability and willingness to recommend mHealth apps in hypertension management

**Fig. 1:**
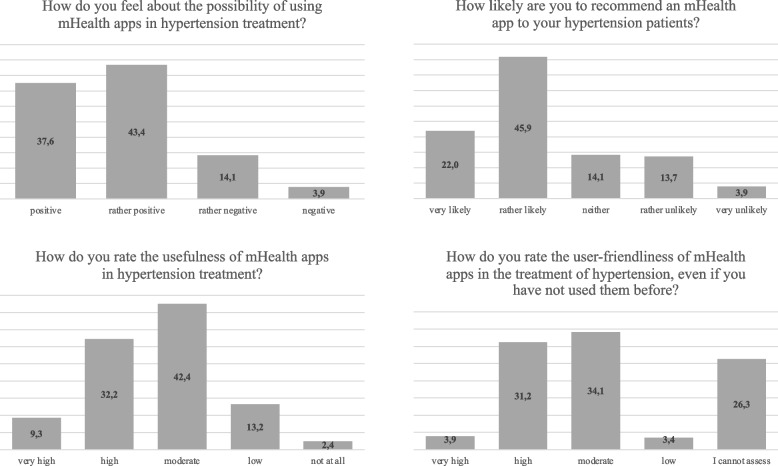
Illustrations of the attitudes, perceived usefulness, usability and willingness to recommend mHealth apps in hypertension management; figures in %; *n* = 205

The attitude of respondents towards the inclusion of mHealth apps in hypertension treatment is predominantly positive. Overall, 81% (166/205) of GPs are positive or rather positive about the inclusion of mHealth apps in treatment (37.6% (77/205) and 43.4% (89/205), respectively). A minority of 18% (37/205) are rather negative or negative (14.1% (29/205) and 3.9% (8/205), respectively). Regarding the likelihood of recommending an mHealth app to patients with hypertension, the physicians indicate a high propensity: 22.0% (45/205) consider it very likely and 45.9% (94/205) consider it somewhat likely that they would recommend a mHealth app to their patients. However, 14,1% (29/205) of respondents are neutral and a small proportion, 13,7% (28/205) think it is rather unlikely and 3,9% (8/205) think it is very unlikely. A total of 41.5% of physicians (87/205) rated the usefulness of mHealth apps in hypertension treatment as either high or very high. Specifically, 32.2% (66/205) selected “high” and 9.3% (19/205) selected “very high”. Only a small proportion consider the benefit to be low (13.2%; 27/205) or none at all (2.4%; 5/205). 35.1% (72/205) rate the user-friendliness of mHealth apps as very high or high (3.9% (8/205) and 31.2% (64/205), respectively). Similarly, 34.1% (70/205) felt that they could only give them a medium rating, while 3.4% (7/205) rated them as less user-friendly. 26.3% (54/200) of the GP`s do not feel able to rate usability (please see Fig. 1).

A positive attitude towards including an app into hypertension treatment is associated with the likelihood that the person would recommend a hypertension app (G = 0.955, *p* < 0.001, *n* = 203). Physicians who rate the usefulness (G = 0.887, *p* < 0.001, *n* = 204) and the user-friendliness (G = 0.625, *p* < 0.001, *n* = 149) of mHealth apps as very high or high are more likely to recommend a mHealth app. In addition, more positive physicians are more likely to report a (very) high usefulness of hypertension apps in treatment (G = 0.438, *p* < 0.001, *n* = 202). Those physicians who also tended to have a more positive attitude towards hypertension apps were more likely to rate the user-friendliness of hypertension apps as better to be higher (G = 0.523, *p* < 0.001, *n* = 149) (details can be found in Supplementary Material 6).

#### Information needs

Only 12.2% feel well or very well informed about the use of mHealth apps for patients with arterial hypertension (9.8% (20/205) and 2.4% (5/205), respectively). 59.1% did not feel well or not at all well informed (42% (86/205) and 17.1% (35/205), respectively). 27.8% (57/205) answered partially/neither nor. The following table shows the need for information in detail regarding the aspects of the range of mHealth apps, the costs of mHealth apps, the aim of mHealth apps, the benefits for patients, the function of mHealth apps and the effectiveness of mHealth apps (please see Table 2).

**Table 2:** The need for information in detail regarding various aspects (*n* = 205)

	Very high	High	Moderate	Low	Very low	Missing
Range of mHealth apps in hypertension treatment	14.6% (30)	35.1% (72)	33.7% (69)	11.2% (23)	4.9% (10)	0.5% (1)
Costs of mHealth apps in hypertension treatment	17.6% (36)	36.1% (74)	26.3% (54)	11.7% (24)	6.8% (14)	1.5% (3)
Aim of mHealth apps in hypertension treatment	13.7% (28)	37.1% (76)	28.8% (59)	12.2% (25)	6.8% (14)	1.5% (3)
Benefits for patients in hypertension treatment	19.5% (40)	39.5% (81)	22.9% (47)	10.2% (21)	7.3% (15)	0.5% (1)
Function of mHealth apps in hypertension treatment	13.7% (28)	42.4% (87)	27.3% (56)	10.2% (21)	5.9% (12)	0.5% (1)
Effectiveness of mHealth apps in hypertension treatment	20.0% (41)	42.4% (87)	22.0% (45)	9.8% (20)	5.4% (11)	0.5% (1)

Furthermore, a (very) high need for information is associated with an increased likelihood of recommending a hypertension app. Physicians who indicated a very high or high need for information are more likely to recommend the app, particularly regarding app offerings (G = 0.710, *p* < 0.001) and functionality (G = 0.339, *p* < 0.001) (details can be found in Supplementary Material 6).

### Focus groups

#### Participants characteristics

The sample of the two multiprofessional focus groups consisted of a total of 15 participants, including ten women and five men, representing various sectors of the healthcare system. The two focus groups included four nurses, one app developer, three GPs, two health insurance representatives, three cardiologists, and two additional physicians. Participants came from regions with varying population sizes to ensure a broad perspective on the study’s topic. This heterogeneous composition contributed to diverse and well-informed insights from different professional backgrounds and regional contexts (detailed information can be found in Supplementary Material 7). The first focus group lasted 1 h and 28 min and the second focus group lasted 1 h and 27 min.

### Why is the need for information so high?

In medical practices and clinics, there is a lack of systematic and structured communication of information about digital prevention measures such as mHealth apps. This issue affects also medical assistants, who have a central and crucial role in the daily management of medical practices. These healthcare professionals often inform themselves about available apps on their own initiative.

*“We frequently have patients asking about them* [mHealth apps], *and we are honestly still quite inexperienced and need to educate ourselves.”* (FG2J_General Practitioner_male_Family practice)

Some participants expressed that information is often not tailored to specific target groups and that helpful materials are lacking.

*“In practice, everything is still very analog; we use a lot of paper, and there is little that is automatically integrated into the system.”* (FG2G_General Practitioner_female_Health care centre)

Another possible explanation for information deficits in relation to mHealth apps is that the question is not based on a conscious distinction between tacit and explicit knowledge. People often have tacit knowledge about the handling and use of mHealth apps based on their previous experience, which is based on intuitive, experience-based understanding of the general use and benefits of mHealth apps. However, they often lack the explicit or specific knowledge that includes detailed, evidence-based knowledge of specific features, effectiveness, and targeted use of mHealth apps to fully understand and use them effectively.

*“I would like to separate the level of information into implicit and explicit knowledge. I actually think it's quite good and quite nice that I don't have any explicit knowledge in the sense of app XY, because as a doctor who then prescribes it, I would have to consider whether it's not something, to put it badly, driven by the pharmaceutical lobby, that I'm now explicitly recommending one thing without there being any major studies on effectiveness.* [] *But I already have the implicit knowledge that this can be recorded digitally, for example, and what the benefits could be, both for patients and for me, and I can pass this on implicitly, for example.“* (FG1J_Physician_male_Clinic)

#### Why do less informed physicians recommend apps more often than informed ones?

A surprising phenomenon is that physicians with a higher need for information about mHealth apps recommend them more often than their colleagues with a lower need for information. From the stakeholders'perspective, this may be explained by the fact that less informed physicians delegate responsibility to their patients, often believing that patients themselves are better equipped to decide whether an app suits them.

*“I just tell patients: Take a look at it and let me know what you find out.”* (FG2F_Cardiologist_female_Clinic)

This approach often reflects a pragmatic way of handling a topic about which physicians feel uncertain. Less informed physicians tend to overestimate the benefits of apps, viewing them as simple solutions for complex problems. As a result, they may be more likely to recommend apps without addressing their practical challenges. In contrast, informed physicians who have delved deeper into the subject are more aware of the deficiencies, such as lack of interoperability, low adherence, and insufficient compensation. This awareness often leads to a more cautious attitude.

*“When you engage more deeply with apps, you see the gaps and problems – it makes it harder to recommend them in good conscience.”* (FG1J_Physician_male_Clinic)

Some physicians deliberately choose not to actively promote apps because they lack the time and resources to provide comprehensive advice to their patients. In such cases, the responsibility often remains with the patients.

*“We just don’t have the time to dive into all the details, so I often leave it up to the patients to handle it themselves.”* (FG1G_Nurse_femal_Clinic)

#### Proposed solutions to bridge the knowledge gap

During the focus group discussions, participants proposed several practical solutions to bridge the existing information gap around mHealth apps. Illustrative quotes supporting these proposed solutions are presented in Table 3. Participants expressed a need for easily accessible information that would enable physicians and medical assistants to gain a quick overview of the functionalities and effectiveness of mHealth apps. Flyers with QR codes or concise guides could effectively address information gaps. Health insurance companies, professional associations, and app developers should collaborate to promote and disseminate information on mHealth. Such partnerships could increase both reach and acceptance.

**Table 3:** Participant quotes on proposed solutions to bridge the knowledge gap regarding mHealth apps in hypertension management

Subthemes: proposed Solutions to bridge the knowledge gap	Representative quotes
Need for easily accessible information (e.g. flyers with QR codes)	"*I think it would be helpful to have flyers with QR codes that patients can take home to review at their own pace."* (FG2D_Cardiologist_female_Clinic)
Cross-sector collaboration to disseminate information	*"Health insurers could work more closely with other stakeholders, like professional associations or app developers, to disseminate information."* (FG1G_Nurse_female_Clinic)
	*"Another good idea would be to get the German Heart Foundation on board […]. Or with the German Hypertension League […]. That might also be quite helpful."* (FG1E_Nurse_female_Health care centre)
	*"Maybe you can actually get the KVs [Associations of Statutory Health Insurance] involved and say that you can download flyers, information material, whatever, and have them sent to you and then pass them on accordingly."* (FG1D_Nurse_female_Family practice)
Integration of mHealth apps into established disease management programmes	*"This could work similarly to chronic care programmes, where health insurers promote usage and provide a structured framework."* (FG1D_Nurse_female_Family practice)
Importance of promoting intrinsic motivation over traditional information campaigns	*„Yes, above all I would like to polarize a bit, perhaps to heat up the discussion a little. I would like to make the point that, as they say, information campaigns fail. I often see it with older colleagues, too, that they come to it with the expectation that, yes, I should be told about it or I should be trained. And I think that's a problem. Patients need to be self-motivated to use it, and practices need to be self-motivated. And by that I mean intrinsic self-motivation. When in doubt, they have to pick up the offer themselves. And I believe you can achieve this by presenting a vision of benefits. So to train someone, to say, come and listen to this, I don't think that achieves anything in clinical reality. The only thing that helps is to tell them that it has these benefits, this is the shared vision that we have, this could be the impact, this is the story that we tell here, why we are doing this.“* (FG1J_Physician_male_Cinic)
Need for collaborative implementation among different stakeholders	*"I believe that it really is a big interplay between everyone afterwards. […] Everyone has to work well together, and everyone must be prepared to take the time to explain this to the patient."* (FG1G_nurse_female_Clinic)

Participants suggested integrating apps into existing programmes, such as disease management programmes, which are already well-established in Germany and considered successful. However, the focus group discussion also expressed a critical attitude towards traditional information campaigns and training approaches and emphasized the need to promote intrinsic self-motivation among both patients and health care professionals in order to increase the use of mHealth apps. Pure information transfer is considered to be less effective in clinical reality. Instead, it is suggested to communicate a vision of benefits by demonstrating concrete advantages and a common goal that can serve as motivating factors for both groups. The implementation and use of mHealth apps requires a collaborative interaction between various stakeholders. It is emphasized that all parties involved—from medical assistants and physicians to health insurance companies and mHealth specialists—must work together and be willing to invest time in explaining and communicating to patients. Responsibility does not lie with a single party, but is shared by all stakeholders.

#### Mixed-methods integration

The mixed-methods design enabled a comprehensive exploration of general practitioners’ information needs and attitudes toward mHealth apps in hypertension care. Several types of data interference were observed between the quantitative and qualitative strands. In many areas, findings were convergent: both data sources highlighted a high demand for information on effectiveness, usability, and reimbursement, as well as a generally positive, yet cautious, attitude toward app recommendations. At the same time, the two strands complemented each other: while digital competence was rated as high in the questionnaire, focus group participants often expressed uncertainty in their actual use of digital tools. Qualitative insights also helped explain why certain applications, such as DiGAs, are rarely used in practice, despite being viewed favourably (e.g., due to lack of information or integration into daily workflows). In some cases, discrepancies emerged, such as between optimistic ratings of mHealth usefulness and doubts about real-world implementation.

## Discussion

The aim of this study was to determine the current attitudes towards the use of mHealth apps in hypertension management and the recommendation behaviour of GP´s, as well as to analyse the existing need for information and to determine how this can be fulfilled in order to establish acceptance in daily care.

### Principal results

The present study shows a predominantly positive attitude among GPs towards the integration of mHealth apps in hypertension management. This corresponds with the findings of other studies [11, 12, 27], which also report growing acceptance of digital health technologies among healthcare professionals. However, the results highlight a notable discrepancy between the current recommendation rate of 41.5% and the significantly higher prospective willingness to recommend mHealth apps, at 77.6%. This suggests that the potential of mHealth apps is accepted, but that their actual use in healthcare is often inadequate. Our results show that the willingness to recommend mHealth apps depends on individual attitudes and structural conditions [28]. We have shown that positive attitudes towards the integration of mHealth apps into hypertension treatment correlate significantly with the willingness to recommend these mHealth apps to others. Perceived usefulness and user-friendliness also play a decisive role, as GPs who reported high levels of usefulness and user-friendliness are more likely to recommend mHealth apps [29–31]

The majority of GPs would recommend an mHealth app to their patients. This finding is consistent with other studies in which GPs were involved [10–12, 32–34]. The results of our study align with a physician survey conducted by the German health insurance company BARMER [34] in 2020. The results of the survey revealed that approximately half of the physicians viewed mHealth apps positively and would recommend or prescribe them during patient consultations. However, around 60% of respondents reported feeling poorly prepared to advise patients on mHealth app use. In a recent study [35], in which 2,160 physicians in Germany were surveyed, 37% stated that they would prescribe DiGAs (digital health applications). This nearly twofold increase within two years suggests a growing willingness among physicians to adopt digital health technologies. Whether a similar trend exists for the recommendation of non-certified mHealth apps remains unclear and warrants further investigation to better understand the acceptance and potential application of these tools. It is also conceivable that digital technologies will increasingly become part of routine care as a response to the ongoing shortage of specialised care. The positive attitudes toward mHealth apps observed in this study could reflect the challenges currently facing healthcare providers, including worsening resource constraints, increasing workloads, and growing demands on day-to-day clinical practice. In this context, physicians may view digital technologies as valuable tools to help manage workloads, streamline processes, and maintain high-quality care, potentially explaining their favourable outlook on mHealth app integration. Additionally, the impact of COVID-19 must be considered as a significant catalyst for both positive attitudes toward and increased use of digital technologies. Studies indicate that the pandemic fostered a more favourable outlook on digital health technologies, as their use often became a necessity during this period [36].

Another key finding of this study is the remarkable lack of information among respondents about mHealth apps. More than half of the participants do not feel sufficiently informed about mHealth apps in hypertension treatment. Studies have already shown that the lack of specific knowledge about the functioning and benefits of mHealth apps is a major barrier to their acceptance and integration into daily practice [12, 37–40]. Further education and specific training could therefore be a crucial step in promoting the use of these technologies [40]. At the same time, our focus groups offer approaches that contribute to an understanding of the current situation and identify specific solutions. We were able to show that tacit knowledge plays a central role in the use of mHealth apps. In surveys, we often focus on explicit knowledge; however, this does not correspond to people's actual knowledge levels. Tacit knowledge is a valuable but often neglected resource when dealing with mHealth apps. In order to exploit the full potential of mHealth apps in everyday health care routines, it is necessary to systematically identify tacit knowledge and make it visible in order to empower health care professionals.

Future research should investigate the relationship between information needs and the utilization of mHealth app potential, particularly exploring whether insufficient information needs might indeed influence app recommendation behaviours. Nevertheless, the focus group discussions also highlighted that structured information dissemination is desired by healthcare professionals. This indicates a need for tailored strategies to improve the integration and use of health apps in clinical practice.

### Practical implications

The results of the study underline the need for a more precise understanding of information needs in order to effectively meet the requirements of the various stakeholders in the healthcare sector. One important aspect is the identification of the specific knowledge that users need in order to use mHealth apps effectively. The theory proposed by Nonaka and Takeuchi [41] describing the transition from tacit to explicit knowledge, provides a valuable framework for addressing knowledge gaps in the use of mHealth apps. Targeted interventions such as structured training programmes specifically addressing digital health technologies in hypertension management, coaching programmes or the introduction of digital navigators [42] can transform tacit knowledge often being based on experience, into explicit knowledge that is accessible for wider application. A shift in roles, where physicians actively engage in testing and familiarizing themselves with mHealth apps, could substantially enhance the acceptance and seamless integration of these technologies. Providing test access could allow physicians to engage hands-on with these applications and thus develop a deeper understanding of their functions. This combination of structured knowledge transfer, hands-on experience and explicit knowledge dissemination could form the basis for an effective and sustainable implementation of mHealth apps in clinical practice. One practical consequence for the implementation of mHealth apps is better knowledge transfer. Audience-specific resources could play a decisive role in better informing healthcare professionals and patients about the use and benefits of these technologies. Examples include flyers with QR codes that facilitate access to detailed information, or short manuals and training videos that convey relevant content in a practical and understandable way. Sharing positive practice examples and case reports could help foster confidence and uptake among healthcare providers. In addition, cooperation between health insurers, medical professional associations and app developers should also be established to provide comprehensive information about the possible uses and benefits of mHealth apps. There are already approaches to involving physicians in mHealth app development [43]. Such a coordinated strategy would not only close the knowledge gaps, but also strengthen confidence in these technologies and facilitate their integration into everyday clinical practice. Another approach is to facilitate the integration of mHealth apps into the existing workflows of medical practices in order to reduce the administrative and manual workload on health professionals. A continuous flow of information on new digital health offerings and their benefits is necessary to keep healthcare providers up to date. Systematic communication of scientific evidence into practice should support healthcare professionals in making evidence-based decisions regarding the use of digital health technologies. Independent assessments, recommendations, and certifications could further support evidence-based use of mHealth apps and facilitate their integration into routine care. App developers should also consider providing concise, practice-relevant update information to users to ensure healthcare providers remain informed about new features and improvements. Another demand is a reimbursement for the use and consultation of mHealth apps. From a policy perspective, our findings highlight the need for systematic information infrastructures and support mechanisms to ensure that primary care physicians can access up-to-date, validated knowledge on available mHealth apps. Policymakers could leverage existing frameworks, such as disease management programmes, and collaborate with professional societies and statutory health insurance associations to promote informed adoption of mHealth technologies in routine care. For practice, promoting hands-on experience with selected apps and fostering a culture of critical, yet open, digital engagement within primary care teams could help close the current knowledge gap. In the long term, the establishment of mHealth apps within structured frameworks such as disease management programmes is a promising option. By integrating these apps into already existing programmes, they could be utilised more effectively.

### Limitations

The study has several limitations that should be taken into account when interpreting the results. Firstly, the use of self-completed questionnaires carries the possibility of self-selection bias. In addition, the study was conducted exclusively in one region of Germany, which may limit the generalizability of the results to other regions or countries with different healthcare systems, digital infrastructure or cultural attitudes towards digital health technologies. Although the study targeted GPs and offered a financial incentive of EUR 50 to encourage participation, this incentive may have influenced participation rates or the nature of responses, leading to potential response bias. The sample may not fully represent the diversity of GPs in Germany, particularly those practicing in rural or underserved areas. In addition, the study relies on self-reported data, which is prone to recall error and social desirability bias; participants may overstate their positive attitudes or understate the challenges they face in adopting mHealth apps. Although the questionnaire was pre-tested to ensure clarity and relevance, there is still a risk that the questions may be interpreted differently by participants, which could affect the consistency and reliability of the data collected. It should also be noted that around 50% of the physicians in the sample of the questionnaire are under the age of 50. Another methodological limitation concerns the qualitative content analysis of the focus groups. Although coding was performed independently by two researchers, no formal calculation of inter-rater reliability was conducted. The results of our study may therefore skew more positive, as younger physicians are generally more familiar with digital technologies compared to their older counterparts. This observation is supported by other studies, which have demonstrated that younger physicians tend to exhibit a more positive attitude toward digital technologies than older physicians [10, 12]. Another limitation of the study is the potential selection bias in the recruitment of the focus group participants, who likely volunteered due to a specific interest in mHealth apps. This self-selection may have resulted in the overrepresentation of individuals with greater knowledge and a more favourable attitude toward mHealth apps in the discussions. Additionally, there is a risk of social desirability bias within the focus groups, as participants may have provided responses they perceived as"correct"or aligned with expectations, rather than expressing their genuine opinions.

## Conclusions

Our results emphasize the large discrepancy between the theoretical potential of mHealth apps and their practical implementation in everyday life. Although GPs in Germany are generally positive about the use of mHealth apps in the treatment of arterial hypertension, there are significant obstacles to widespread adoption. Future research should focus on the interaction between knowledge levels and mHealth app recommendation behaviour to promote effective implementation of mHealth apps in clinical practice.

## Supplementary Information

Supplementary file 1. 

Supplementary file 2.

Supplementary file 3. 

Supplementary file 4. 

Supplementary file 5. 

Supplementary file 6.

Supplementary file 7. 

## Data Availability

All data relevant to the study are included in the article or uploaded as supplementary information. For further questions regarding the reuse of data, please contact the corresponding author (susann.may@mhb-fontane.de).
