# Supplementary material for: Bridging the knowledge gap: a mixed-methods study on general practitioners’ information needs for mHealth apps in hypertension treatment in Germany
Source: BMC Health Serv Res. 2025 Sep 10;25:1195. doi: 10.1186/s12913-025-13192-9 (PMC12421746; doi:10.1186/s12913-025-13192-9)
Supplement: Supplementary file 3 [file 12913_2025_13192_MOESM3_ESM.pdf]

Supplementary Material File 3

Good Reporting of a Mixed Methods Study (GRAMMS) checklist

Guideline	Section: page
Describe the justification for using a mixed methods approach to the research question	Methods: 4
Describe the design in terms of the purpose, priority and sequence of methods	Methods: 4-7
Describe each method in terms of sampling, data collection and analysis	Methods: 4-7
Describe where integration has occurred, how it has occurred and who has participated in it	Methods: 4-7
Describe any limitation of one method associated with the present of the other method	Limitations: 19-20
Describe any insights gained from mixing or integrating methods	Results: 12-16

O'Cathain A, Murphy E, Nicholl J. The quality of mixed methods studies in health services research. J Health Serv Res Policy. 2008;13: 92-98.
