# Supplementary material for: Bridging the knowledge gap: a mixed-methods study on general practitioners’ information needs for mHealth apps in hypertension treatment in Germany
Source: BMC Health Serv Res. 2025 Sep 10;25:1195. doi: 10.1186/s12913-025-13192-9 (PMC12421746; doi:10.1186/s12913-025-13192-9)
Supplement: Supplementary file 4 [file 12913_2025_13192_MOESM4_ESM.pdf]

Supplementary Material File 4

Part I - In the first part of the survey, we will ask you general questions about digital prevention measures. The following statements refer to innovations in general practice. Please check the answer that applies to you most.

The following is general health-related information from the Internet and your personal assessment. The statements do NOT refer to hypertension care. Please select the statement that applies to you. The results of this question are not presented in the manuscript.

	Strongly agree	Agree	Neutral	Disagree	Strongly disagree
I know how to find helpful health resources on the Internet.	O	O	O	O	O
I know how to use the Internet to answer my health questions.	O	O	O	O	O
I know what health resources are available on the Internet.	O	O	O	O	O
I know where to find helpful health resources on the Internet.	O	O	O	O	O
I know how to use the health information I find on the Internet to help me.	O	O	O	O	O
I have the skills I need to evaluate the health resources I find on the Internet.	O	O	O	O	O
I can tell high quality from low quality health resources on the Internet.	O	O	O	O	O
I feel confident in using information from the Internet to make health decisions.	O	O	O	O	O

Do you use or recommend the following digital services or would you recommend them to your patients? The results of this question are not presented in the manuscript.

	Do you currently use this digital service in your everyday practice?			Would you recommend this digital service?	
	Yes	No, never before	No, but before	Yes	No
Video consultation with patients	O	O	O	O	O
Communication via email with patients	O	O	O	O	O
Online appointment booking (e.g., via Doctolib)	O	O	O	O	O
Other (optional): ____________________	O	O	O	O	O

	Do you currently use this digital service in your everyday practice?			Would you recommend this digital service?	
	Yes	No, never before	No, but before	Yes	No
Live-streamed online course (e.g., fitness course via video with live participation and interaction with the trainer)	O	O	O	O	O

Recorded online course (e.g., on-demand fitness course via video without interaction)	O	O	O	O	O
Telephone health consultation (e.g., nutrition counseling, smoking cessation)	O	O	O	O	O
General health apps (e.g., step counter, Google Fit)	O	O	O	O	O
Digital health applications (DiGA) (e.g., DiGA ProHerz)	O	O	O	O	O
Disease-specific apps (not DiGAs) (e.g., for hypertension, diabetes)	O	O	O	O	O
Wearables (e.g., fitness watches, smartwatches)	O	O	O	O	O
Symptom checker (e.g., a diagnostic app like Ada)	O	O	O	O	O
Websites with health information (e.g., gesundheitsinformationen.de, hochdruckliga.de)	O	O	O	O	O
Videos on health topics (e.g., on YouTube)	O	O	O	O	O
Other (optional): ____________________	O	O	O	O	O

Part II - In the next part of the survey, we will ask you questions about mHealth apps that can be used in the treatment of arterial hypertension (e.g. Hypertonie.App or Cardio Coach).

How well informed do you feel about the use of mHealth apps for patients with arterial hypertension?

Very well	Well	Neither	Not well	Not well at all
O	O	O	O	O

How much information do you need regarding the following aspects?

	Very high	High	Moderate	Low	Very Low
Range of mHealth apps in hypertension treatment	O	O	O	O	O
Costs of mHealth apps in hypertension treatment	O	O	O	O	O
Aim of mHealth apps in hypertension treatment	O	O	O	O	O
Benefits for patients in hypertension treatment	O	O	O	O	O
Function of mHealth apps in hypertension treatment	O	O	O	O	O
Effectiveness of mHealth apps in hypertension treatment	O	O	O	O	O

What information would you still like to receive regarding mHealth apps in hypertension treatment? (free text answer)

____

How do you feel about the possibility of using mHealth apps in hypertension treatment?

Positive	Rather positive	Rather negative	Negativ
O	O	O	O

How likely are you to recommend a mHealth app to your hypertension patients?

Very likely	Rather likely	Neither	Rather unlikely	Very unlikely
O	O	O	O	O

How do you rate the usefulness of mHealth apps in the treatment of arterial hypertension?

Very high	High	Moderate	Low	Very low
O	O	O	O	O

How do you rate the user-friendliness of mHealth apps in the treatment of arterial hypertension, even if you have not used them before?

Very high	High	Moderate	Low	I cannot assess
O	O	O	O	O

What benefits do you see in the use of apps for hypertension care? The results of this question are not presented in the manuscript.

	Fully agree	Agree	Partly agree	Disagree somewha t	Strongly disagree
Promotion of patient autonomy	O	O	O	O	O
Support in coping with illness	O	O	O	O	O
Increase in patient safety	O	O	O	O	O
Improved medication adherence	O	O	O	O	O
Support in healthcare delivery	O	O	O	O	O
Positive impact in the doctor-patient relationship	O	O	O	O	O
Other (optional): _______________________________	O	O	O	O	O

What barriers do you see in the use of apps for hypertension care? The results of this question are not presented in the manuscript.

	Fully agree	Agree	Partly agree	Disagree somewha t	Strongly disagree
Data protection concerns regarding the security of patients' health data	O	O	O	O	O
Legal uncertainties regarding physician liability risks	O	O	O	O	O
Lack of information on the range of m-Health apps	O	O	O	O	O
Lack of knowledge regarding the specific effectiveness	O	O	O	O	O
Increased workload for practice staff in integrating m-Health apps into daily practice routines	O	O	O	O	O
Additional workload due to additional tasks in patient care	O	O	O	O	O
Additional effort regarding onboarding and training	O	O	O	O	O
Inadequate remuneration for physicians	O	O	O	O	O
Technical challenges due to different systems and interfaces	O	O	O	O	O
Negative impact on doctor-patient communication	O	O	O	O	O
m-Health apps are not suitable for all patients	O	O	O	O	O
m-Health apps are not yet fully developed	O	O	O	O	O
Other (optional): _______________________________	O	O	O	O	O

Part III - Vignettes - The results of this question are not presented in the manuscript.

Part IV - In the following we ask you some socio-demographic questions. We would like to point out once again that the survey is anonymous, i.e. no conclusions can be drawn about your person.

Hoe old are you?	How long have you been working as a doctor?
______ Years	______ Years

Which gender would you classify yourself as?

female	male	non-binary
O	O	O

In which federal state do you mainly work?

____ (federal state)

How many inhabitants does the place where you mainly work have?

<5.000	5.000 - 20.000	20.001 – 100.000	100.001 – 1 Mio.	>1 Mio
O	O	O	O	O

Are you employed and/or self-employed?

employed	self-employed	both
O	O	O

Wo arbeiten Sie überwiegend?

Single handed practice	Practice partnership	Medical care center	Hospital	Other:
O	O	O	O	O
