# Supplementary material for: Bridging the knowledge gap: a mixed-methods study on general practitioners’ information needs for mHealth apps in hypertension treatment in Germany
Source: BMC Health Serv Res. 2025 Sep 10;25:1195. doi: 10.1186/s12913-025-13192-9 (PMC12421746; doi:10.1186/s12913-025-13192-9)
Supplement: Supplementary file 5 [file 12913_2025_13192_MOESM5_ESM.pdf]

Supplementary Material File 5

Excerpt Interview Guide DiPaH Focus groups

Research questions: There is a great need for information on digital prevention measures for arterial hypertension, particularly with regard to the use of apps in hypertension care. How can this situation be explained? How should information on reach, impact, costs, etc. ideally be communicated?

Guiding question/ narrative impulse	Check aspects	Concretizing questions	Maintenance and control questions
The results of the questionnaire survey show that there is a huge need for information about mHealth apps. How do you explain this?	**Information status/ information transfer**	How well informed do you feel about digital prevention measures and mHealth apps for arterial hypertension?<br><br>What sources of information do you currently use?<br><br>Which channels and formats do you prefer for providing information (e.g. training courses, webinars, specialist journals)?<br><br>Why do less informed physicians recommend apps more often than informed ones?<br><br>Do you have any suggestions on how the provision of information could be improved?	
