# Supplementary material for: Bridging the knowledge gap: a mixed-methods study on general practitioners’ information needs for mHealth apps in hypertension treatment in Germany
Source: BMC Health Serv Res. 2025 Sep 10;25:1195. doi: 10.1186/s12913-025-13192-9 (PMC12421746; doi:10.1186/s12913-025-13192-9)
Supplement: Supplementary file 6 [file 12913_2025_13192_MOESM6_ESM.pdf]

Supplementary Material 6

Table Supplementary Material 6. Goodman and Kruskal's gamma between mHealth app recommendation and information needs (in general, mHealth app offerings, cost of mHealth apps, objective of mHealth apps, benefit for patients, functioning of mHealth apps, efficacy of mHealth apps).

	Gamma-Value	Significance level
A greater general need for information and a higher likelihood of recommending an mHealth app	0.301	< 0.001
A greater need for information about mHealth app offerings and a higher likelihood of recommending an mHealth app	0.710	< 0.001
A greater need for information about the costs of mHealth apps and a higher likelihood of recommending an mHealth app	0.425	< 0.001
A greater need for information about the objectives of mHealth apps and a higher likelihood of recommending an mHealth app	0.544	< 0.001
A greater need for information about the benefits of mHealth apps and a higher likelihood of recommending an mHealth app	0.543	< 0.001
A greater need for information about how mHealth apps function and a higher likelihood of recommending an mHealth app	0.617	< 0.001
A greater need for information about the efficacy of apps and a higher likelihood of recommending an mHealth app	0.564	< 0.001
