# Supplementary material for: Bridging the knowledge gap: a mixed-methods study on general practitioners’ information needs for mHealth apps in hypertension treatment in Germany
Source: BMC Health Serv Res. 2025 Sep 10;25:1195. doi: 10.1186/s12913-025-13192-9 (PMC12421746; doi:10.1186/s12913-025-13192-9)
Supplement: Supplementary file 7 [file 12913_2025_13192_MOESM7_ESM.pdf]

Supplementary Material 7

Table Supplementary Material 7. Focus groups participants sample characteristics.

	Profession	Duration of professional acitivity	Age in years	Gender	Number of inhabitants	Setting
FG1E	Nurse	10	29	female	5.000-20.000	Health care center
FG1F	Nurse	1	22	female	<100.00	Clinic
FG1I	Head of Product Strategy & Innovation	8	29	male	<100.00	Startup
FG1D	Nurse	12	32	female	5.000-20.000	Family practice
FG1K	General practitioner	3	34	female	>5.000	Joint practice
FG1J	Physician	3	30	male	5.000-20.000	Clinic
FG1H	Physician	14	42	male	<100.00	Clinic
FG1G	Nurse	10	34	female	<100.00	Clinic
FG2D	Cardiologist	22	48	female	5.000-20.000	Clinic
FG2I	Health insurance company representative	21	42	male	<100.00	Health insurance company
FG2J	General practitioner	10	40	male	>5.000	Family practice
FG2F	Cardiologist	17	43	female	<100.00	Clinic
FG2H	Sports and health consultant	4	23	female	<100.00	Health insurance company
FG2B	Cardiologist	13	37	female	<100.00	Clinic
FG2G	General practitioner	9	38	female	<100.00	Health care center
